# Effects of computer-assisted oral anticoagulant therapy

**DOI:** 10.1186/1477-9560-10-17

**Published:** 2012-08-30

**Authors:** Rune Skovgaard Rasmussen, Pernille Corell, Poul Madsen, Karsten Overgaard

**Affiliations:** 1 University Hospital of Copenhagen, Herlev Hospital, Copenhagen, Denmark; 2 University Hospital of Copenhagen, Gentofte Hospital, Copenhagen, Denmark; 3 University Hospital of Copenhagen, Frederiksberg Hospital, Copenhagen, Denmark; 4 Copenhagen Experimental Stroke Unit, University of Copenhagen, Panum Institute 12-2-34, Blegdamsvej 3, DK-2200, Copenhagen, Denmark

## Abstract

**Background:**

Computer-assistance and self-monitoring lower the cost and may improve the quality of anticoagulation therapy. The main purpose of this clinical investigation was to use computer-assisted oral anticoagulant therapy to improve the time to reach and the time spent within the therapeutic target range compared to traditional oral anticoagulant therapy by physicians.

**Methods:**

54 patients were randomized equally into 3 groups. Patients in two groups used CoaguChek^®^ systems to measure international normalized ratio (INR) values and had dosages of anticoagulation treatment calculated in a computer system by an algorithm specific to each group. The third group received traditional anticoagulation treatment by physicians. The obtained INR values were compared regarding the time to reach, and the time spent within, the therapeutic target range, corresponding to INR values from 2 to 3.

**Results:**

Patients randomized to computer-assisted anticoagulation and the CoaguChek^®^ system reached the therapeutic target range after 8 days compared to 14 days by prescriptions from physicians (*p* = 0.04). Time spent in the therapeutic target range did not differ between groups. The median INR value measured throughout the study from all patients by CoaguChek^®^ at 2.5 (2.42–2.62) was lower than measured by a hospital-based Clinical and Biochemical Laboratory at 2.6 (2.45–2.76), (*p* = 0.02).

**Conclusions:**

The therapeutic target range was reached faster by the use of computer-assisted anticoagulation treatment than prescribed by physicians, and the total time spent within the therapeutic target range was similar. Thus computer-assisted oral anticoagulant therapy may reduce the cost of anticoagulation therapy without lowering the quality. INR values measured by CoaguChek^®^ were reliable compared to measurements by a clinical and biochemical laboratory.

## Background

Oral anticoagulant therapy with vitamin K antagonists is increasingly used and has widely documented effects for prophylaxis and treatment of several thromboembolic events [1,2]. The number of Danish patients on oral anticoagulant therapy is increasing and although the exact number is unknown, about 1.6% of all Danes are estimated to receive oral anticoagulant therapy [2-4]. Oral anticoagulant therapy increases the risk of bleeding and if the treatment is not strictly controlled, bleedings diminish the net benefit of therapy [1-5]. In some clinical studies the annual risk of serious bleedings reached 6-7%, with 1% fatal [1,6-9]. The time in therapeutic target range (TTR), corresponding to an international normalized ratio (INR) between 2 and 3, correlates strongly and negatively with the incidence of bleedings and thromboembolic events and is often used as a surrogate marker for efficacy and quality of the therapy [1,2,7,8,10].

The establishment of specialized anticoagulation clinics has improved oral anticoagulant therapy among a large number of patients by standardizing procedures [11], and the use of computer-aided-management-systems reduce the amount of time used by the physician prescribing oral anticoagulant therapy and improve the quality of the therapy [12-15].

Oral anticoagulant therapy is a troublesome and demanding task for both patients and physicians. Self-management of oral anticoagulant therapy using coagulometers at home is increasingly used and is safe [14-16]. The quality of oral anticoagulant therapy should be continuously documented and monitored to ensure maximum efficacy and a minimum of complications, and especially improving strategies for maintaining INR between 2 and 3 are important to reduce hemorrhagic or thromboembolic events [10].

The main purpose of this clinical investigation was to use computer-assisted oral anticoagulant therapies to improve the time to reach and the time in the TTR compared to traditional oral anticoagulant therapy by physicians. Specifically our primary endpoint was to maintain INR in the TTR for at least 80% of the treatment time, since specialized anticoagulation clinics already have been able to maintain INR in the TTR during 60 to 70% of the treatment time, although traditional therapies by physicians may result in less than 50% treatment time in the TTR [14]. Finally we examined if INR measured by patients using CoaguChek^®^ correlated with the INR measured at our hospital-based Clinical Biochemical Laboratory.

This investigation is innovative in combining two technological advancements (computerized treatment algorithm and point of care testing) in patients on warfarin with the implicit ultimate goal of streamlining and improving dosing regimens.

Oral direct thrombin inhibitors are rapidly emerging as a possible therapeutic option for oral anticoagulation without the need for intensive dose/monitoring regimens, but oral direct thrombin inhibitors may be expensive, and cannot be used in patients with impaired renal function, and therefore warfarin treatments are not easily replaced, especially in patients were warfarin is well-tolerated, adding to the current relevance of this clinical investigation [17].

## Methods

A computer-aided management system for telemedical oral anticoagulant therapy named CoaguTel was developed and tested cooperatively by the IT-company Context and Frederiksberg Hospital (FH). This computer system contained an electronic patient record and enabled continuous monitoring of the quality of oral anticoagulant therapy. CoaguTel offered a computer-aided management system for the physician regarding indications, contraindications, recommended therapeutic INR intervals and the recommended duration of the treatment. The system contained algorithms for dosages, e.g. when an INR value was obtained, the system suggested both a dosage of warfarin (Marevan^®^) and a time point for obtaining the next INR. INR values were automatically transferred from the Clinical Biochemical Laboratory to CoaguTel.

The patient population in this investigation was included January 20^th^ 2002 to January 6^th^ 2004 from both FH’s Cardiological-Endocrinological Department and the Stroke Unit. Most of the patients were warfarin naïve and had ischemic stroke with atrial fibrillation, atrial fibrillation without stroke and other diagnoses e.g. deep venous thrombosis or pulmonary embolism. Patients with indications for oral anticoagulant therapy were offered to participate in this study and there were no exclusion criteria. Cognition was not measured due to limited resources, but all patients were screened to make sure that they were able to participate in the study and were thought to be compliant to anticoagulant therapy. Patients who after learning and under guidance were able to operate the CoaguChek® system were offered to use this system, but all INR values measured by the CoaguChek® system were simultaneously measured by the Clinical Biochemical Laboratory. None of the patients refused to participate, and patient data were recorded in CoaguTel system. Due to the use of traditional oral anticoagulant therapy by a physician it was not possible to blind investigators completely, but investigators were blinded for group-specific algorithms (group 1 and 2) and all statistical analysis of results were performed by personnel who did not participate in data collection, study design and who had no competing interest. Furthermore the randomization of patients was randomly performed by computer stratified according to age and diagnosis/indication for oral anticoagulant therapy to one of the following three treatment groups:

Group 1  Computer-assisted oral anticoagulant therapy using an algorithm-based initiation followed by a specially designed algorithm (FH algorithm) to calculate maintenance dosages [6,12].

Group 2  Computer-assisted oral anticoagulant therapy using an algorithm-based initiation (identical to Group 1) and a modified Hillingdon algorithm to calculate maintenance dosages [6,18].

Group 3  Traditional oral anticoagulant therapy maintained by a physician without computer assistance.

The algorithm-based initiation of the anticoagulation treatment was as described in Additional file 1; although patients estimated as needing a low dosage for maintenance during the first 4 days received 1.5 tablets a day instead of 2 (dosage at initiation × 0.75), but hereafter patients were treated corresponding to Additional file 1 with all dosages multiplied by 0.75. Twelve or 15 days after initiation the computer-assisted patients continued receiving maintenance dosages either by the FH or the Hillingdon algorithm.

The Hillingdon-algorithm was: M = 1 + (0.4 + (1 / (D + 5)) * ln (T / A), where ”M” is the dosage ratio corresponding to the factor, by which the previous dosage should be multiplied with in order to reach the next dosage. “D” is the number of days between two INR values, while “T” is the INR target value and “A” is the current INR value [18].

The FH algorithm was a slight adaptation of the maintenance-algorithms of Table 1. The purpose of the modification was to make a functional algorithm also for other therapeutic intervals than those in Table 1.

**Table 1:** The FH-algorithm

**Adjusting the Oral Anticoagulation Treatment**			
2.0–3.0	Therapeutic INC interval		2.5–3.5
**INR**	**Acute treatment**	**Maintenance dosage**	**INR**
>10	Give Vitk with or without FFP. Pause VKA until INR is within therapeutic interval (2- > 7 days)	Reduce to 50% or more	>10
6.0–10	Pause VKA for 2–3 days. Vitk may be administered	Reduce 30–40%	7.0–10
5.0–5.9	Pause VKA for 1–2 days	Reduce 20–30%	5.5–6.9
3.5–4.9	Pause VKA for 0–1 day	Reduce 10–20%	4.0–5.4
3.1–3.4	None	Reduce 0–10%	3.6–3.9
2.0–3.0	None	No change	2.5–3.5
1.7–1.9	None	Increase 0–10%	2.1–2.4
1.5–1.6	Double dosage of VKA 1 day	Increase 20–30%	1.7–2.0
<1.5	Double dosage of VKA 1 day. Heparin may be administered	Increase 40–50%	<1.7

The FH-algorithm was a modification to the algorithm used in this scheme (6). VitK: Vitamin K_1_. FFP: Freshly frozen plasma. VKA: Vitamin K- antagonist. The suggested changes to the maintenance dosages presume a steady state corresponding to an unchanged dosage of warfarin for more than 1 week or phenprocoumon for more than 1 month, and that the sensitivity for VKA is unchanged in the following period of time.

By using the FH algorithm the computer used the following algorithms:

a. If INR was in the TTR (A = T): No adjustment of dosage.

b. If INR was above the TTR (A > Ul, where Ul = upper limit of the TTR), then the following dosage adjustments were made: Fw = 0.3 + 0.67 Ul/A, (Fw was the dosage ratio and corresponded to the factor, by which the last maintenance (weekly) dosage was to be multiplied with to provide the next weekly dosage). Furthermore, 1) if Ul/A < 0.88, then treatment with Marevan^®^ was paused for 24 hours, 2) if Ul/A < 0.61, then Marevan^®^ was paused for 48 hours, 3) if Ul/A = 0.5–0.3, then Marevan^®^ was paused for 72 hours and 4) if Ul/A < 0.3, then Marevan^®^ was paused until obtaining the next INR value.

c. If INR was below the TTR (A < Ll, where Ll = Lower limit of the TTR), then the following dosage adjustments were made: Mn = 2.3–1.3 × A/Ll, although the maximum accepted value of Mn was 1.5. Mn was the factor, which the previous maintenance dosage was multiplied with to provide the next maintenance dosage. If A/Ll < 0.8, then a double dosage of Marevan^®^ was administered at the day of the INR measurement.

Two blood samples were obtained simultaneously from patients randomized to computer-assisted management. A venous blood sample was sent to the Clinical Biochemical Laboratory for measurement of INR and patients used the coagulometer CoaguChek^®^ S (Roche Diagnostics, Switzerland) to measure INR. The dosage of Marevan^®^ was calculated using the INR values obtained from CoaguChek^®^. Patients’ randomized to traditional oral anticoagulant therapy had INR measured at the Clinical Biochemical Laboratory and dosages of Marevan^®^ were calculated by different physicians at the clinical departments of FH.

### Ethics

This clinical investigation was subject to the guidelines of the 2nd Helsinki Declaration. The ethics committees of Copenhagen and Frederiksberg approved the investigation, file no. 01-014/02. All patients provided written consent in order to participate. Patients were only allowed to participate by their own free will and could cancel their consent for participation at any time point. The database was approved by the relevant Danish authorities.

### Statistical analysis

Data from all patients were collected as INR values and dosages related to the INR values. This investigation had insufficient data to provide valid measurements of unwanted side effects like thromboembolic events and severe bleedings. Calculations of the time in the TTR were performed according to the method of Rosendaal [19], where the total observation time for each patient is categorized in classes of INR values. Additionally this method presupposes a linear correlation between two neighboring INR values. Thereafter the total time in oral anticoagulant therapy was divided into time in, above or below the TTR, and the results are provided as percentages [19].

The data was not distributed normally and therefore non-parametric statistics were used. Mann-Whitney’s test was performed when comparing individual groups (unpaired observations) followed by the Kruskal–Wallis nonparametric analysis of variance test to test for overall significant differences among groups. For the time in, above or below the TTR, Wilcoxon’s tests were used for paired analyses. Wilcoxon’s test was further used to evaluate differences between INR values from patients, who had INR measured twice the same day, in order to compare the measurements from the Clinical Biochemical Laboratory and the CoaguChek^®^.

P-values below 0.05 were considered statistically significant. Median results are displayed as values followed by the associated 25^th^ and 75^th^ percentiles in brackets, unless a more elaborate description is provided.

### Power analysis and sample size calculations

In order to compare treatment efficacies, a change in the time spent in the TTR from 50% to 80% would require 14 patients in both the computer-assisted and the traditionally treated groups, if Z_2_-alpha and Z-Beta (type 1 and type 2 errors) were 5% and 50% respectively. Thus a total of 28 patients would need to be included in two groups. We chose to add a third group in order to compare two different algorithms to traditional therapy. It may be noted that investigators have found that physicians were able to maintain patients in the TTR for 30 to 60% of the treatment time [14], and using 30% for reference instead of 50%, sizes would have been reduced to 5 patients per group in this study.

## Results

54 patients were included in this investigation. 19 patients were randomized to the FH algorithm, 18 patients to the modified Hillingdon algorithm, and 17 patients to traditional oral anticoagulant therapy performed by physicians. The patients were investigated during an average of 28 weeks, and the total time of treatment was 29 years and 4 months. Twenty-three (43%) of the patients were women and 31 (57%) were men. There were no significant differences in age, gender or the total treatment duration between the three treatment groups (Table 2).

**Table 2:** Descriptive characteristics of patients

**Characteristics**	**Hillingdon-algorithm**	**FH-algorithm**	**Traditional anticoagulation therapy**
Number of patients	18	19	17
Age, years*	70 (56–78)	68 (57–80)	69 (61–76)
Gender, %			
Women	44	42	41
Men	56	58	59
Treatment duration, days*	117 (79–416)	123 (78–491)	97 (64–243)

*Median values with associated 25–75 percentiles.

**Figure 1:**
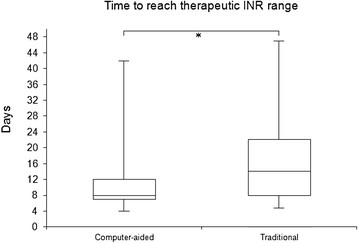
**Patients assigned to computer-assisted oral anticoagulant therapy reached the TTR faster than patients assigned to traditional oral anticoagulant therapy performed by a physician.** Median days until first INR measurements in the TTR are illustrated in combination with associated 25^th^ and 75^th^ percentiles in respective groups. Error bars define 5^th^ and 95^th^ percentiles. ^*^P < 0.05.

Patients randomized to computer-assisted oral anticoagulant therapy reached TTRs at day 8 (7–12) (median and 25–75 percentiles) compared to traditional oral anticoagulant therapy performed by physicians, where TTRs were reached at day 14 (8–22) (*p* = 0.04), as shown in Figure 1.

The time in percent, when the different groups were in, above or below the TTR, and the highest recorded INR values, can be seen in Table 3.

**Table 3:** Basic results of INR measurements

**INR measurements**	**Hillingdon-algorithm**	**FH-algorithm**	**Traditional anticoagulation therapy**
% time in INR range	49 (33–63)	55 (50–65)	55 (49–66)
% time in INR range < 2	26 (15–34)	23 (16–42)	31 (23–37)
% time in INR range > 3	23 (14–38)	13 (0–28)*	7 (0–18)**
Highest recorded INR	4.0 (3.4–4.7)	3.4 (2.9–5.1)	3.3 (2.9–4.2)

FH: ”Frederiksberg Hospital” algorithm. Median values with 25–75 percentiles. *P < 0.05 and **P < 0.01 compared to Hillingdon-algorithm.

A small difference was found comparing INR values measured by the Clinical Biochemical Laboratory and by CoaguChek^®^, with higher INR values measured by the Clinical Biochemical Laboratory, 2.60 (2.45–2.76) versus, 2.50 (2.42–2.62), (*p* = 0.02) measured by CoaguChek^®^. More than 94% of all INR values obtained the same day from the same patient and from both the Clinical Biochemical Laboratory and CoaguChek^®^ were within 0.5 INR-units.

## Discussion

In this investigation, computer-assisted oral anticoagulant therapy was at least as efficient as traditional oral anticoagulant therapy performed by physicians, and therefore computer-assisted oral anticoagulant therapy may provide a more cost-effective solution.

Authors of a recent meta-analysis of oral anticoagulation trials found that keeping patients within INR 2 to 3 should minimize risks of both hemorrhages and thromboembolic formations, and that it was safer to keep patients between INR 3 and 4 than below 2 [10]. We did not achieve significant results, but tendencies indicated that our used algorithms were at least as efficient in avoiding INR values below 2 as physicians (Table 2). By maintaining patients in the TTR for ~ 50% of the treatment time the use of algorithms did not expose patients to higher risks of either hemorrhagic or embolic complications than traditional therapy.

Our goal of keeping patients in the TTR for at least 80% of the treatment time was not reached indicating that the algorithms were insufficiently designed to meet this endpoint, but the use of algorithms made patients reach the TTR faster than by traditional therapy.

We observed a difference between INR values measured by CoaguChek^®^ and by the Clinical Biochemical Laboratory. The difference was not larger than the expected variance when comparing two Danish laboratories and the difference did not provide any significant influence upon the dosing of warfarin. Therefore INR values obtained and measured by CoaguChek^®^ were reliable compared to analysis by a laboratory [20-22].

In our present investigation, computer-assisted oral anticoagulant therapy was at least as efficient in maintaining patients in the TTR as traditional treatments by physicians, thereby potentially eliminating the need for oral anticoagulant therapy maintained by physicians, and strengthening similar evidence provided by other investigators [14,15].

Variability in patients’ genotypes and clinical information may cause an increased risk of hemorrhage and demonstrates the difficulty in warfarin dosing [23]. Our algorithms did not include genotyping as a parameter for warfarin dose calculations, and inclusion of pharmacogenetic algorithms may increase patients’ time in the TTR and reduce adverse events [23].

The mean time in TTR using conventional therapy performed by physicians or medical specialists varied from 30% to 60% in several studies, and when these patients were not in the TTR, 75% of patients were below the range [14]. In our study physicians maintained INR in the TTR for 55% of the treatment time, and when not in range, patients were more likely to be below the range than above. Therefore the performance of physicians in our study corresponded to other studies [14].

Unfortunately the algorithms in our study did not keep patients in the TTR for longer periods of time in comparison to patients receiving traditional anticoagulation therapy. Specialized anticoagulation clinics have maintained patients in TTR for 60% to 70% of the treatment time, although a substantial number of patients still were below or above the TTR for considerable periods of time [14]. Furthermore in many countries visiting specialized anticoagulation clinics may be time consuming for patients or inconvenient thereby reducing patients’ treatment satisfaction and quality of life.

In a recent study patients spent 80% of treatment time in the TTR by twice weekly performing INR measurement at home and online report. In comparison patients in an anticoagulation clinic spent 73% of their treatment time in the TTR [24]. The before mentioned study did not use algorithms for warfarin dose calculations, but had specialized anticoagulation staff (5 medical doctors) reporting new warfarin doses to patients within 4 hours of receiving patients’ self-reported INR values. Thus the latter study did require more manpower than our computerized algorithms, but also provided the possibility for patients to measure INR at home. Thus the major challenge may be to improve algorithms, possible by the inclusion of pharmacogenetics, to provide similar or better anticoagulation management compared to current efficacies of physicians and medical specialists.

An increased risk of adverse events may explain the fact that too few patients with increased risk of thromboembolic events receive oral anticoagulant therapy. Only 12–40% of patients with atrial fibrillation and increased risks of thromboembolic events received anticoagulation treatment. Such findings may be partly explained by contraindications, but often it is a subjective decision by physicians to treat a patient with oral anticoagulant therapy or not [25,26]. In one stroke trial 18% of patients experienced atrial fibrillations and 70% had atrial fibrillations diagnosed before the stroke, but only 21% of these patients received anticoagulation treatment and many thromboembolic events could presumably be avoided by anticoagulation treatment [25]. Oral anticoagulant therapy is demanding for the patient, and an increasing number of patients increase hospital workloads. Self-monitored oral anticoagulant therapy, where a patient measures INR by using a coagulometer at home, decreases the workload of both patients and therapists. Thus our study supports the growing positive evidence of using computer-assisted oral anticoagulation, because the quality and costs of computer-assisted oral anticoagulation therapy were equal or better than traditional oral anticoagulation therapy performed by physicians. Such findings may lead to an increased use of oral anticoagulation therapies and reduce risks of thromboembolic strokes.

## Conclusions and suggestions for future research

Results from our study showed that dosing algorithms matched the performance of physicians, and future optimizations of dosing algorithms, possibly including pharmacogenetics, may provide more time in the TTR, reduce the involvement of physicians or medical specialists in dose calculations and expand the cost-effectiveness of computer-assisted oral anticoagulation therapies.

## Abbreviations

INR: International normalized ratio; TTR: Therapeutic target range.

## Competing interest

There are no competing interests.

## Authors’ contributions

All authors have made substantial contributions to the conception, design, analysis and interpretation of data, and the drafting of the manuscript. All authors have approved the published version of the manuscript.

## Supplementary Material

Additional file 1 ** Computer-assisted algorithms.** Patients assigned to computer-assisted algorithms were treated according to this scheme from day 1 to day 12 or day 15 (6). An example of using the table above: After 4 days of 5 mg daily warfarin treatment (5 mg = 2 tablets) the INR was 2.6. According to point 3, the patient should continue with 1 tablet daily. Day 8 INR was 2.0. According to point 4, the patient should hereafter continue treatment with 8 tablets per week. Point 5 shows that INR should be controlled again at day 15. Click here for file
