# Supplementary material for: Effects of computer-assisted oral anticoagulant therapy
Source: Thromb J. 2012 Aug 30;10:17. doi: 10.1186/1477-9560-10-17 (PMC3502261; doi:10.1186/1477-9560-10-17)
Supplement: Additional file 1 — Computer-assisted algorithms. Patients assigned to computer-assisted algorithms were treated according to this scheme from day 1 to day 12 or day 15 (6). An example of using the table above: After 4 days of 5 mg daily warfarin treatment (5 mg = 2 tablets) the INR was 2.6. According to point 3, the patient should continue with 1 tablet daily. Day 8 INR was 2.0. According to point 4, the patient should hereafter continue treatment with 8 tablets per week. Point 5 shows that INR should be controlled again at day 15. [file 1477-9560-10-17-S1.pdf]

Additional file 1

1.	**Tablets/day, day 1-4:**	2				
2.	**INR, day 5:**	<1,8	1,8 - 2,4	2,5 - 3,0	3,1 - 3,5	>3,5
3.	**Tablets/day, days 5-7:**	2	1,5	1	0,5	0
4.	**INR, day 8:**	The table below shows the estimated maintenance dosages **as tablets per week**				
	<1,8	16	13	10	6	4
	1,8 - 2,4	14	11	8	5	3
	2,5 - 3,0	12	9	7	4	2
	3,1 - 3,5	10	7	5	3	1
	>3,5	8	6	4	2	0

The tablets should be as evenly distributed as possible througout the week

5. Next INR: Day 12 / Day 15

6. Dose adjustements: Use Table 2 for adjusting mantenance dosage
